# Molecular Profiling in Daily Clinical Practice: Practicalities in Advanced Cholangiocarcinoma and Other Biliary Tract Cancers

**DOI:** 10.3390/jcm9092854

**Published:** 2020-09-03

**Authors:** Angela Lamarca, Zainul Kapacee, Michael Breeze, Christopher Bell, Dean Belcher, Helen Staiger, Claire Taylor, Mairéad G. McNamara, Richard A. Hubner, Juan W. Valle

**Affiliations:** 1 Department of Medical Oncology, The Christie NHS Foundation Trust, Manchester M204BX, UK; zainul.kapacee@nhs.net (Z.K.); michael.breeze@nhs.net (M.B.); christopher.bell@nhs.net (C.B.); dean.belcher@nhs.net (D.B.); helen.staiger@nhs.net (H.S.); claire.taylor109@nhs.net (C.T.); Mairead.McNamara@christie.nhs.uk (M.G.M.); richard.hubner@nhs.net (R.A.H.); juan.valle@nhs.net (J.W.V.); 2 Division of Cancer Sciences, University of Manchester, Manchester M204BX, UK

**Keywords:** biliary tract cancer, cholangiocarcinoma, molecular profiling, targeted therapies, FGFR, IDH, mutation, fusion

## Abstract

Background: Molecular profiling is becoming increasingly relevant in the management of patients with advanced cancer; to identify targetable aberrations and prognostic markers to enable a precision medicine strategy. Methods: Eligible patients were those diagnosed with advanced biliary tract cancer (BTC) including intrahepatic (iCCA) and extrahepatic cholangiocarcinoma (eCCA), gallbladder cancer (GBC), and ampullary carcinoma (Amp) who underwent molecular profiling between April 2017 and June 2020 based on analysis of either tumour samples (FoundationOne CDx^®^/Oncomine^®^ platforms) or ctDNA (FoundationOne Liquid^®^ platform (Foundation Medicine, Cambridge, MA, USA)). Baseline patient characteristics and molecular profiling outcomes were extracted. The primary aim was to describe sample failure rate. Secondary aims included description of reason for sample failure, summary of findings derived from molecular profiling, and assessment of concordance between paired tissue and ctDNA samples. Results: A total of 149 samples from 104 individual patients diagnosed with advanced BTC were identified and eligible for this analysis: 68.2% iCCA, 100% advanced stage; 94.2% received palliative therapy. The rate of sample failure was 26.8% for tissue and 15.4% for ctDNA; *p*-value 0.220, predominantly due to insufficient (defined as <20%) tumour content in the sample (the reason for 91.2% of tissue sample failure). Of the 112 samples successfully analysed, pathological molecular findings were identified in the majority of samples (88.4%) and identification of pathological findings using ctDNA, was possible regardless of whether the patient was on active treatment at time of blood acquisition or not (*p*-value 1.0). The rate of targetable alterations identified was 40.2% across all successfully-analysed samples (39 iCCA; 6 non-iCCA): IDH1 mutations (19.1% of individual patients), FGFR2 alterations (10.1% and 5.6% of individual patients had FGFR2 fusions and mutations, respectively); 10.6% of all patients (12.4% of patients with successfully analysed samples) entered trials with matched targeted therapies as a consequence. Concordance of findings for paired tissue and paired tissue-ctDNA was high (3/3; 100% and 6/6; 100%, respectively). Twelve ctDNA samples were taken prior to palliative treatment initiation, median maximum mutant allele frequency (MAF) was 0.47 (range 0.21–19.8); no significant association between reported maximum MAF and progression-free survival (PFS) or overall survival (OS) (all Cox regression *p*-values > 0.273). A total of 15 patients (16.6%) harboured alterations in DNA damage repair (DDR) genes; when treated with platinum-based chemotherapy, there was a trend towards increased partial response rate (21.4% vs. 15.9%; *p*-value 0.653), radiological benefit rate (64.3% vs. 36.2%; *p*-value 0.071), and longer OS (median OS 20.4 months (95% CI 7.9–26.7) vs. 13.3 (95 CI 11.0–16.4); Cox Regression HR 0.79 (95% CI 0.39–1.61), *p*-value 0.527). Conclusions: Molecular profiling is of use for identification of novel therapeutic strategies for patients with advanced BTC (mainly iCCA). One in four archived tissue samples may have insufficient tumour content for molecular profiling; ctDNA-based approaches may overcome these obstacles.

## 1. Introduction

Biliary tract cancers (BTC) represent 3% of all gastrointestinal malignancies, and their incidence (mainly for intrahepatic cholangiocarcinoma (iCCA)) is increasing [1]. Unfortunately, despite advances in adjuvant strategies [2,3], the prognosis remains poor for these patients [4,5], and palliative chemotherapy is the treatment of choice for patients with advanced disease [6,7,8]. 

Molecular profiling of advanced cancer is becoming increasingly relevant in order to tailor therapeutic approaches; not only to identify prognostic markers but also to identify targetable aberrations that could benefit from precision medicine strategies with targeted therapies. Biliary tract cancers are not an exception and even though current development of precision medicine strategies are mainly focused on patients diagnosed with iCCA, it is expected that novel therapies will also be available for extrahepatic cholangiocarcinoma (eCCA), gallbladder cancer (GBC), and ampullary tumours (Amp) in the coming future [9]. 

The development of targeted therapies is significantly impacting the care and management of patients with advanced iCCA [9,10], for whom inhibitors of fibroblast growth factor receptor (FGFR) fusions [11,12,13,14,15,16,17,18,19,20,21,22] and isocitrate dehydrogenase (IDH) mutations [12] are becoming a reality. Alternative potential targets for future “Precision Medicine” strategies in BTC may include chromatin remodeling genes (*ARID1*, *BAP1,* and *PBRM1*) and other aberrations such as BRAF and RNF43 mutations, HER2 and HER3 amplifications or NTRK fusions [10,23,24,25,26,27]. The potential clinical implications of molecular profiling are not limited to the used of targeted therapies [28]; the identification of DNA damage repair aberrations have been associated with better responses to platinum-based chemotherapy strategies and could therefore enable tailoring of chemotherapy choice [29,30,31]. There is also data supporting the role of immunotherapy for patients with mismatch repair deficient tumours [32,33].

Despite recent advances, there are still significant challenges: 

Firstly, patients with BTC identified as having a targetable aberration following molecular profiling still represent a small proportion and is mainly limited to those with an iCCA diagnosis. Thus, the discovery of new targets and development of new molecules is needed and efforts should be focused not only on iCCA but also on other BTCs. 

Secondly, cancers arising from the biliary tree are known for being technically challenging to biopsy [34]. Although there are less often issues obtaining samples for confirmatory pathological diagnosis of BTC, it may be difficult to obtain sufficient tumour samples for molecular profiling [35]. This may necessitate repeat biopsies, incurring the associated procedure risk [36]. 

Sample failure rate for BTCs and related reasons have not been widely described, as these patients are usually not included in reported series [37]. To facilitate access to molecular profiling in the absence of available tumour tissue for analysis, liquid biopsy strategies, such as circulating tumour deoxyribonucleic acid (ctDNA), have been shown to be a potentially more accessible method of identifying actionable genomic alterations. This has resulted in more timely application of genotype-matched therapies in other malignancies, such as lung cancer [38]. Alternative studies in cholangiocarcinoma, assessing the concordance between blood ctDNA and tissue DNA reported concordance of 74% (92% for patients with iCCA) [39]. Good concordance (92%) for IDH1 mutation identification between tumour tissue and ctDNA was also reported in the ClarIDHy phase III trial, exploring the role of ivosidenib in IDH1-mutant iCCA [40]. Whilst acknowledging that there are currently limitations to this technology, the use of ctDNA for molecular profiling of patients without an easily-biopsiable lesion could be beneficial for future practice [41,42,43]. 

The clinical relevance of molecular profiling for patients diagnosed with advanced BTC (mainly iCCA) is widely accepted. However, practical challenges faced such as sample failure rate, the reasons for this and potential solutions, such as the use of liquid biopsies (ctDNA) have not been widely discussed in advanced BTC and will be explored in this study.

## 2. Materials and Methods

All consecutive patients diagnosed with advanced BTC, including iCCA, eCCA, GBC, and Amp, who underwent molecular profiling at our institution between April 2017 and June 2020 were eligible. Patients provided written informed consent for molecular profiling to be performed; in addition the retrospective analysis of these data was approved by our institutional Audit Committee (approval number 19/2634).

Clinical baseline characteristics and demographic data, were collected. Cancer-related systemic treatment information (chemotherapy schedule, line of treatment, radiological response, and disease progression) was retrieved from electronic records for the line of therapy relevant to the sample acquisition time. Partial response was defined as per Response Evaluation Criteria in Solid Tumors (RECIST) v.1.1; radiological benefit was defined as those scenarios in which a reduction in size of marker lesions was reported, regardless of whether partial response criteria as per RECIST v1.1. was met or not.

Molecular profiling using either tumour tissue or blood (ctDNA) was included. Platforms employed for tumour sample testing included FoundationOne CDx^®^ and/or Oncomine^®^; ctDNA was analysed using the FoundationOne Liquid^®^ platform (Cambridge, MA, USA). In addition to the presence/absence of pathogenic aberrations and fusions, data on mutant allele frequency (MAF), microsatellite instability (MSI) and tumour mutational burden (TMB) were collected whenever reported. The Oncomine^®^ platform (ThermoFisher Scientific, Shanghai, China) assessed fusion on ribonucleic acid (RNA), and whether the quality control for this analysis was successful or not was also captured. Targetable findings were those for whom potential treatment strategies were available, such as, but not limited to, FGFR2 fusions or mutations, IDH1 mutations, and HER2/3 amplifications. Where pathogenic relevance of amplifications was not determined in the provided report (i.e., Oncomine^®^ platform), amplified genes with copy number variation (CNV) of 3–5 were classified as “unknown significance”, while CNV of >5 were classified as pathogenic. The presence or absence of variants of unknown significance was also collected. 

The primary aim was to describe the sample failure rate. Secondary aims included reason for sample failure, description of findings derived from molecular profiling (i.e., clinical implications, impact on patient outcome or response to treatment). Additionally, concordance rate was reported for patients with matched tissue analysed with two different platforms or for patients with a paired tissue and ctDNA sample. Data for patients who had ctDNA analysed immediately prior to initiation of systemic therapy were explored separately to evaluate the impact of ctDNA findings (maximum MAF among others) on patient outcome. Finally, patients with mutations in any gene associated with DNA damage repair (DDR) were analysed separately, including baseline characteristics, clinical outcome and radiological response to platinum-based chemotherapy, compared to patients without alterations in DDR.

Descriptive statistical analysis using STATA v.12 (College Station, TX, USA, https://www.stata.com/stata12/) was performed. Progression-free survival (PFS) was defined as the time between initiation of treatment until disease progression; if the patient died without documented radiological progression, date of death was used as time of progression (unless death was considered to be non-cancer related). Both overall survival (OS) from the time of sample acquisition (OSsample) and from the time of systemic treatment initiation (OStreatment) were calculated. Patients free of progression or death at the time of last data update, were censored at the date of last follow-up for PFS and OS, respectively. Chi-Square test, Fisher’s exact test, T-test and logistic regression were used, when appropriate. Survival analysis was undertaken using Kaplan Meier, Log-Rank test and Cox Regression. 

## 3. Results

A total of 149 samples from 104 individual patients were included in this analysis. 

### 3.1. Patient Characteristics

A total of 149 samples from 104 individual patients were identified as eligible for this analysis. Most patients (68.2%) had a diagnosis of iCCA; all (100%) had advanced disease at the time of sample analysis. The majority of patients started some form of palliative therapy (*n* = 98; 94.2%), mainly cisplatin and gemcitabine (92.0%) as first-line therapy (96.0%). Additional details regarding clinical characteristics of these patients are provided in Table 1 (“All patients” column). 

### 3.2. Sample Characteristics and Failure Rate

**Figure 1:**
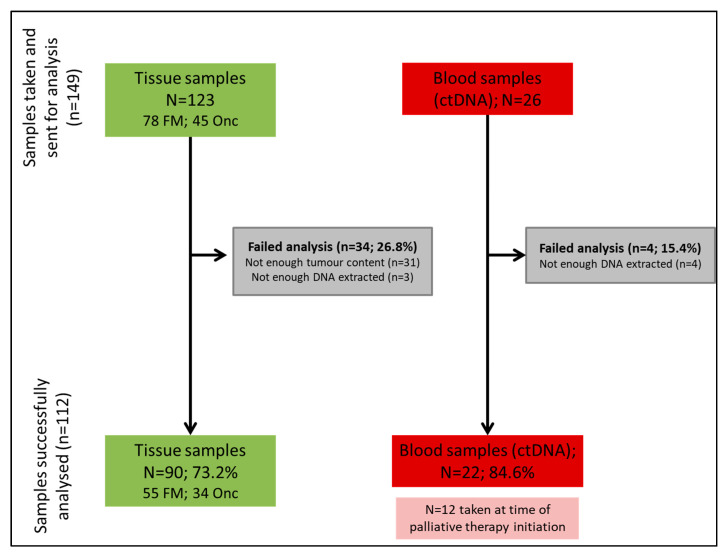
Sample flow. Of the total of 149 samples analysed, 26.8% of the tissue samples and 15.4% of the blood samples failed analysis. Out of the 104 individual patients included, 81 had tumour sample only analysed (no ctDNA analysis performed) (1 tumour sample analysed in 66 patients; >1 tumour sample analysed in 26 patients); 12 patients had ctDNA analysed only (no tumour sample analysed) (1 ctDNA sample analysed in 21 patients; >1 ctDNA sample analysed in 2 patients); in 11 patients, a minimum of 1 tumour and 1 ctDNA samples were analysed. FM: refers to FoundationOne CDx^®^ panel; Onc: refers to Oncomine^®^ panel; *n*: number; %: percentage; ctDNA: circulating tumour DNA; and DNA: Deoxyribonucleic acid.

A summary of sample flow is provided in Figure 1. 

Of 149 samples, 123 were tumour tissue (*n* = 78 FoundationOne CDx^®^, 45 Oncomine^®^) and 26 were blood (all FoundationOne Liquid^®^). The majority of samples were analysed as part of an ongoing clinical trial (114; 76.5%); the median time between sample acquisition and molecular profiling was 7.4 months (range 0–48.7) for archival tissue samples. Baseline characteristics of patients undergoing tumour sample or ctDNA analysis varied significantly, mainly in terms of primary tumour and other characteristics (Table S1): of all the tumour samples analysed, 70.7% were from patients with a diagnosis of iCCA, while 57.7% of ctDNA samples were from patients with iCCA (*p*-value 0.038). 

A total of 112 samples from 89 individual patients were successfully analysed (90 tissue samples (55 FoundationOne CDx^®^, 34 Oncomine^®^)) and 22 ctDNA samples. The rate of sample failure was 24.8% when all samples were analysed together (26.8% for tissue (29.5% FoundationOne CDx^®^, 24.4% Oncomine^®^)) and 15.4% for ctDNA samples; *p*-value 0.220). Even though there was a trend towards longer mean time between sample acquisition and testing in those samples which failed testing (13.2 vs. 9.2 months; *p*-value 0.0615); differences did not reach statistical significance (odd ratio (OR) 1.04 (95% CI 0.99–1.08); *p*-value 0.066). The reasons for failed sample analysis included: 31 tissue samples had insufficient tumour representation (defined as <20% of tumour content); 7 had too low DNA extracted for analysis (3 tumour samples; 4 blood samples). Thus, 31/34 (91.2%) of tissue samples failed due to insufficient tumour representation. Of the 34 tissue samples that were successfully analysed with Oncomine^®^, quality control for RNA fusions failed in 12 (35.3%).

### 3.3. Pathological Molecular Findings 

The summary of the main findings derived from molecular profiling is provided in Table 2. Of the 112 samples successfully analysed, the majority of samples (88.4%) were able to identify presence of pathological molecular findings, both in tissue and ctDNA (88.9% and 86.4%, respectively; *p*-value 0.740). Capacity to identify pathological findings using ctDNA was regardless of whether patients were on active treatment at the time of blood acquisition or not (14/17, 82.4% for patients who were not on treatment at time of blood acquisition vs. 5/5, 100% for patients who were on treatment at time of blood acquisition; *p*-value 1.0). The rate of targetable alterations identified was 40.2% when successfully-analysed samples were accounted for; 45.5% for all samples with pathological molecular alterations (48.8% and 31.8%, for tissue and ctDNA samples, respectively; *p*-value 0.177). A total of 67.9% of samples had at least one variant of unknown significance identified. Of the seven samples analysed for MSI status, all samples were defined as MSI stable. Tumour mutational burden (TMB) was assessed in 7 samples; the median TMB was 2 Mutations/Megabase (Mut/Mb) (range 0–6).

When analysis was solely focused on pathological molecular findings (Table 3) the most frequent alterations identified were TP53 mutations (24.7% of individual patients with at least 1 successfully-analysed sample), KRAS mutations (27.0% of individual patients with at least 1 successfully-analysed sample), followed by IDH1 mutations (19.1% of individual patients with at least 1 successfully-analysed sample), CDKN2A mutations (12.4% of individual patients with at least 1 successfully-analysed sample), and FGFR2 alterations (10.1% and 5.6% of individual patients with at least 1 successfully-analysed sample had FGFR2 fusions and mutations, respectively). 

### 3.4. Clinical Implication of Pathological Molecular Findings

**Figure 2:**
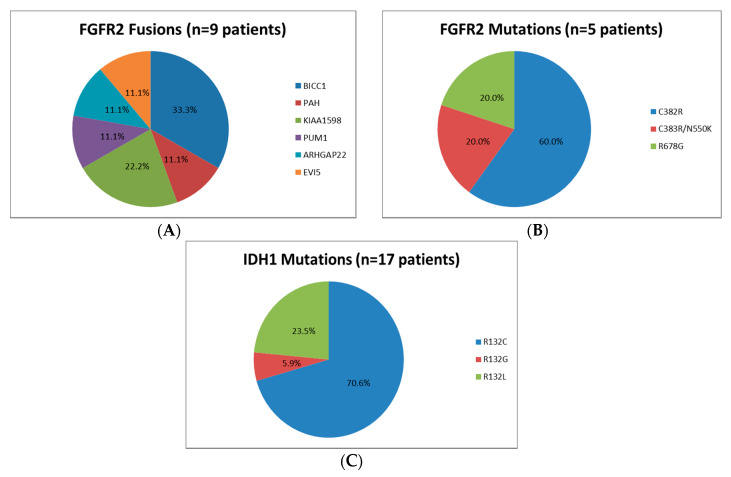
Description of FGFR2 and IDH1 alterations identified. Data for individual patients is provided in Table S3. (**A**): FGFR2 fusions; (**B**): FGFR2 mutations; and (**C**): IDH1 mutations.

The main targetable findings were IDH1 mutations and FGFR2 fusions, present in 19.1% and 10.1% of patients successfully analysed. FGFR2 mutations were identified in 5.6% of individual successful-analysed patients. The presence of other targetable aberrations was identified in <6% of individual patients each (Table 3). Figure 2 provides detail regarding the FGFR2 and IDH1 alterations identified for individual patients. As expected, multiple FGFR2 fusion partners were identified, despite a predominance of BICC1. The most frequent IDH1 mutation reported was C382R. Presence of BAP1 mutations were noted in two patients with FGFR2 mutations and four patients with FGFR2 fusions. The coexistence of IDH1 and FGFR2 aberrations was described in three patients. Neither the presence of IDH1 mutations nor FGFR2 fusions impacted on OSsample (Cox Regression *p*-value > 0.1).

**Figure 3:**
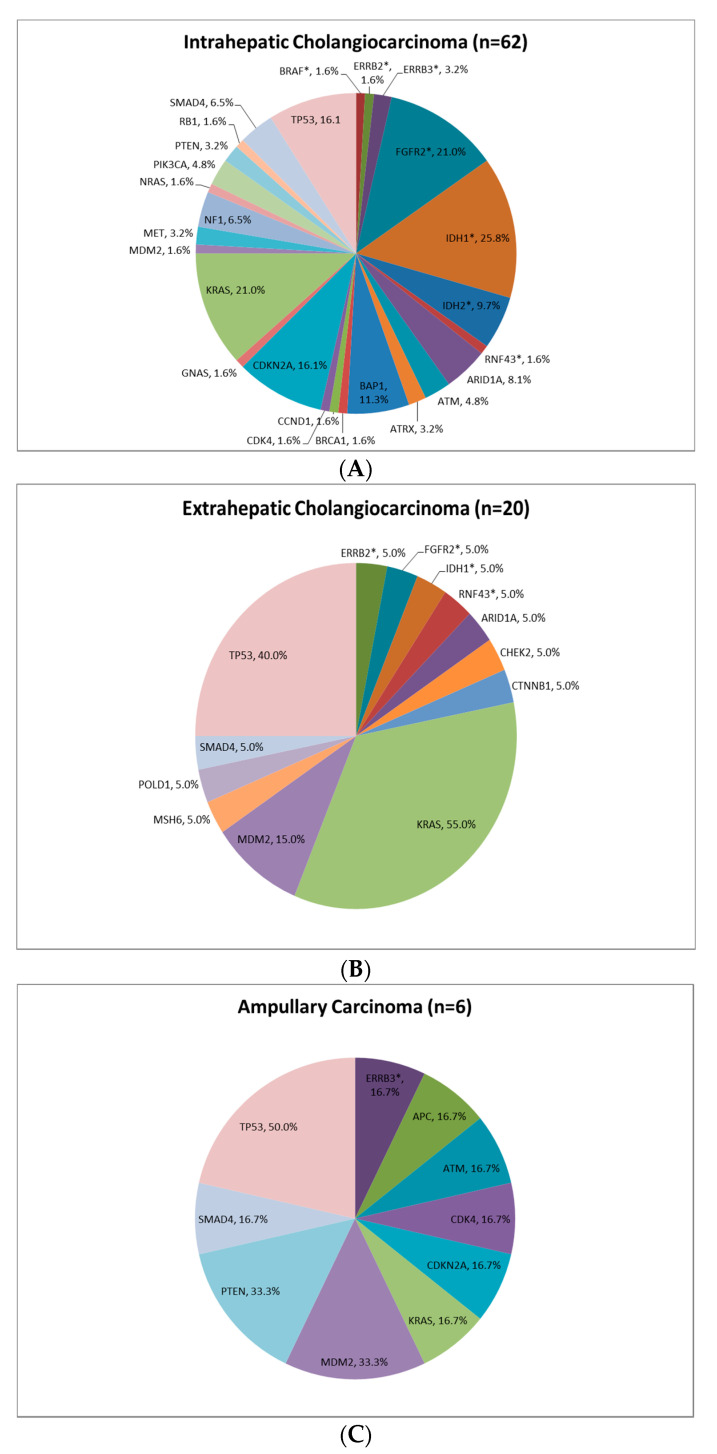
Molecular alterations by primary tumour site. Targetable findings were mainly identified in patients diagnosed with intrahepatic cholangiocarcinoma. * highlight potentially “targetable” alterations in biliary tract cancer. See Tables S2 and S3 for further details on full breakdown of findings and individual patient data, respectively. (**A**): Intrahepatic Cholangiocarcinoma (*n* = 62); (**B**): Extrahepatic Cholangiocarcinoma (*n* = 20); and (**C**): Ampullary Carcinoma (*n* = 6).

Identified alterations by primary tumour type are summarised in Figure 3 and Tables S2–S6. Targetable findings were mainly identified in tumours from patients diagnosed with iCCA (Figure 3A; Tables S2 and S3). Out of the samples with targetable findings (*n* = 45), 39 were iCCA (86.7%) and only 6 were non-iCCA (13.3%). Only 3% of tumours from patients with eCCA and 8% of ampullary tumours were identified to have a HER-2 (Figure 3B; Tables S2 and S4) and HER-3 alterations (Figure 3C; Tables S2 and S6), respectively. Only 1 patient’s tumour with GBC was successfully analysed (Table S5), in whom an ALK fusion was identified. 

As a direct result of the pathological molecular findings, 11/104 patients (10.6% of the whole population; 11/89 (12.4%) of patients with successfully analysed samples) entered a clinical trial with a targeted therapy (7 with FGFR inhibitors, and 4 with IDH inhibitors); all of whom were patients diagnosed with iCCA. 

### 3.5. Analysis of Paired Tissue Samples

Twenty-six patients had matched tissue analysed by two different tissue technologies; 10 samples failed; thus, matched findings were available for 13 patients (Table S7). There was adequate concordance in identification of targetable findings such as FGFR2 alterations and IDH1/IDH2 mutations (concordance 3/3; 100%); one patient with an FGFR2 fusion identified in one of the panels was not included as the fusion quality control failed in the second panel, and thus fusions were not tested for. Concordance in relation to other prevalent (but non-targetable) genes such as KRAS was lower (4/7). 

### 3.6. Analysis of Paired Tissue and ctDNA Samples

Table 3 provides a summary of pathological molecular findings by sample type. Rate of individual gene aberrations reported in tissue and ctDNA vary when the whole series is analysed. However, it is most likely that this is reflection of differences within baseline characteristics of the cohort of patients undergoing ctDNA or tissue analysis (mainly in regards to primary tumour site, within others (Table S1). 

**Figure 4:**
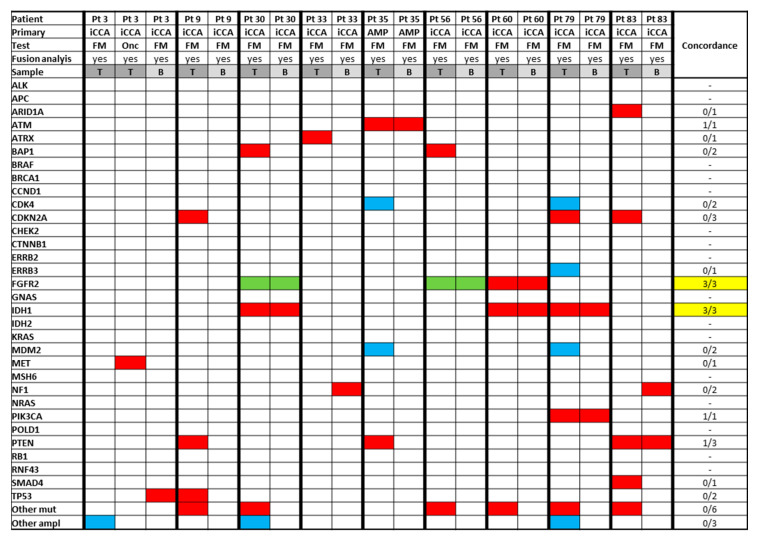
Paired tissue and ctDNA samples. iCCA: intrahepatic cholangiocarcinoma; eCCA: extrahepatic cholangiocarcinoma; GBC: gallbladder cancer; Amp: ampulla of Vater carcinoma; FM: Foundation Medicine^®^, refers to FoundationOne CDx^®^ or FoundationOne Liquid^®^ panel; Onc: Oncomine^®^ panel; B: blood (ctDNA); T: tumour tissue; %: percentage; Pt: patients ID. Red box: mutation; blue box: amplification; green box: fusion; and yellow box: targetable alteration.

In order to analyse concordance between paired tissue and ctDNA samples, patients with such data available (nine patients with successfully-analysed samples) were separately assessed (Figure 4). 

Of these nine patients, three patients, two patients, and one patient were identified to have an IDH-1 mutation, FGFR-2 fusion, and FGFR-2 mutation in the tumour sample, respectively. All these alterations were confirmed on ctDNA analysis (concordance 6/6; 100% for identification of these targetable findings).

### 3.7. ctDNA Analysed Prior to Palliative Therapy Initiation

Of 24 samples of ctDNA available, ctDNA was successfully analysed prior to the initiation of systemic therapy in 12 individual patients. For this subgroup, baseline characteristics are provided separately in Table 1 (“ctDNA prior to treatment cohort” column). Only one patient (8.3%) was receiving palliative chemotherapy at time of ctDNA sample acquisition, which did not preclude identification of ctDNA and adequate analysis. There was a predominance of female (83.3%) patients with advanced iCCA (66.7%). Five, five, and two patients started palliative therapy as a first-, second-, and third-line therapy, respectively; most (66.7%) in the form of cisplatin and gemcitabine chemotherapy. At the end of follow-up (median follow-up 8.4 months; range 5.1–11.3), 83.3% and 58.3% of patients had progressed and died, respectively. The estimated median PFS was 4.6 months (95% CI 2.4–8.7). 

Pathological molecular findings in ctDNA were identified in 10/11 patients (90.9%), (Table S8), with a median maximum MAF of 0.47 (range 0.21–19.8). There was no significant association between reported maximum MAF and PFS or OS (all Cox regression *p*-value > 0.273). The most frequently identified pathological molecular alterations were TP53 mutations (58.3%), IDH1 mutations (16.7%), FGFR2 mutation/fusion (16.7%), NF1 mutations (16.7%), and ALK fusion (8.3%) (Table S8), accounting for targetable findings in 5 of the 12 patients (41.7%) (4 iCCA and 1 GBC). 

### 3.8. DNA Damage Repair Genes

Of the 89 patients with successfully-analysed samples, 15 (16.6%) had at least one pathogenic mutation in a DDR gene (Table S9). The most frequently mutated genes in these 15 patients were ATM (26.6%) and BAP1 (46.6%). Baseline characteristics were compared between patients with and without mutations in DDR genes (Table S9), with no significant differences identified between both cohorts. There was a trend towards longer OS in patients with DDR mutations (vs. DDR wild-type) (OSsample: median 20.0 months (95% CI 8.6-not reached) vs. 13.5 (95% CI 10.0–14.9); Cox Regression HR 0.66 (95% CI 0.31–1.39), *p*-value 0.272).

**Figure 5:**
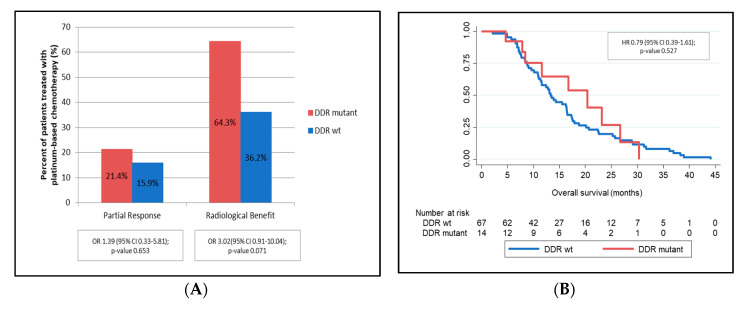
Impact of the presence of DDR gene mutations on outcomes of patients treated with platinum-based chemotherapy. OR: odds ratio; HR: hazard ratio; CI: confidence interval; %: percentage; and wt: wild-type. (**A**): Partial Response and Radiological Benefit; (**B**): Overall survival.

Of these 89 patients, 81 were treated with platinum-based chemotherapy (14 with mutations in DDR genes). Median PFS did not vary significantly in the presence or absence of DDR gene mutations; Cox Regression HR 0.82 (95% CI 0.41–1.66), *p*-value 0.585 (Table S9). However, those patients treated with platinum-based chemotherapy harbouring DDR mutations (vs. DDR wild-type) showed a trend towards improved partial response rate (21.4% vs. 15.9%), radiological benefit (64.3% vs. 36.2%) and OS benefit (OStreatment: median 20.4 months (95% CI 7.9–26.7) vs. 13.3 (95% CI 11.0–16.4); Cox Regression HR 0.79 (95% CI 0.39–1.61), *p*-value 0.527) (Figure 5, Supplementary Materials 6).

## 4. Discussion

This study provides an overview of the current challenges of delivering molecular profiling in clinical practice for patients diagnosed with advanced BTC. Tissue sample failure rate is relatively high, with 1 in 4 samples failing analysis, mainly due to insufficient archival tumour content. This failure rate may be overcome by performing ctDNA-based molecular profiling, especially when concentrating on known alterations of interest, such as IDH1 and FGFR2. Unfortunately, patients deriving benefit from molecular profiling were almost exclusively patients diagnosed with iCCA, which highlights the importance of identifying novel targets and developing new drugs for patients with other BTC primary sites of origin. This study also demonstrates that molecular profiling can identify a subpopulation of patients with mutations in DDR genes, who may derive more benefit from platinum-based chemotherapy or other therapeutics targeting the DDR pathway [30].

The failure rate was high for tissue samples (26.8%), regardless of the platform employed and seems unlikely to be associated with archived tissue storage time. The main reason for tissue sample failure was the presence of <20% tumour content for analysis. While this could be minimised by involving a pathologist at the time of selecting tissue blocks, this is most likely to be a reflection of the nature of biopsies acquired in BTCs, and thus a difficult challenge to solve without a repeat biopsy. 

Interestingly, the failure rate for ctDNA was significantly lower (15.4%); even though statistical significance was not reached (*p*-value 0.220) when the failure rate between tissue sample and ctDNA was assessed, likely due to limited power for such comparison and wide 95% CIs. ctDNA sample acquisition is minimally-invasive, and results are clinically meaningful, highlighting that ctDNA may be a reliable way of accessing molecular profiling for patients without sufficient tissue for analysis. In addition, systemic chemotherapy ongoing at the time of blood sample acquisition did not impact on the capacity of ctDNA to identify pathological molecular alterations. The logistics and convenience of ctDNA analysis (easily and immediately accessible blood sample without the need of involving additional departments such as pathology, radiology or endoscopy) compared to challenges of tissue sample analysis may be advantageous, acknowledging the requirement for expertise in analysis and associated cost.

In addition to the above-mentioned benefits favouring ctDNA-based molecular profiling in the absence of sufficient tissue, adequate concordance between tissue and ctDNA findings for patients with paired samples was demonstrated in this study (100% for FGFR2 and IDH1 alterations, which are currently of the most clinical relevance and interest in patients with iCCA in particular). This is in keeping with reported concordance in other studies of 74% (92% for iCCA) [40] and 92% (for IDH1-only) [40], respectively. 

Finally, the potential use of ctDNA as a predictive marker of response to chemotherapy was assessed in the cohort of patients who had ctDNA tested prior to initiation of palliative treatment. There was no impact of higher maximum MAF on PFS, or OS, which may be due to limited sample size within this cohort (12 patients), and exposure to prior chemotherapy in a proportion of the patients included in this cohort. Further studies would be required to assess the potential role of ctDNA in identifying tumour burden, or as a prognostic or predictive tool. ctDNA may also be useful tool to define mechanism of resistance to systemic therapies, if analysed longitudinally. As it stands, ctDNA does not have a diagnostic role and would not replace acquisition of tumour biopsy for pathology confirmation of BTCs.

Almost half of patients (40.2%) had potentially targetable alterations identified, mainly FGFR2 mutations and fusions and IDH1 mutations in patients with advanced iCCA, as expected [9]. Identification of targetable alteration in non-iCCA BTCs was rare and mainly limited to alterations in the HER gene family [9,25,44,45].

These profiling data match other previous observations, such as the co-existence of FGFR2 and BAP1 alterations [46], the episodic co-existence of cases with both fusions/mutations of FGFR2 and IDH1 mutations (which are not mutually exclusive) [47] and BICC1 as the most frequent FGFR2 fusion partners [47,48,49,50,51,52]. A significant prognostic impact for either FGFR2 fusions or IDH1 mutations was not identified, in contrast to prior unconfirmed hypotheses [50,52,53,54].

There was adequate concordance when paired tissue samples were analysed with two different platforms, mainly FGFR2 and IDH1 alterations. Different technologies for assessment of fusions were employed with these two platforms, and a number of samples failed quality check for tissue analysis and therefore resulted in a limited number of observations. However, results did not significantly differ based on the panel employed for tissue analysis. 

A total of 11 patients (10.6%) received genomic-matched targeted therapies (all as part of clinical trials). This highlights another of the challenges faced by treating clinicians: difficulty in accessing treatments for the identified alterations. Obtaining access to non-licensed or non-reimbursed treatment outside the setting of clinical trials is currently one of the main reasons why molecular profiling does not result in a greater influence in clinical management. 

The benefit from molecular profiling may go beyond access to targeted therapies. Mutations in DDR genes were identified in up to 15 patients (16.6%), who, when treated with platinum-based chemotherapy, seemed to derive an increased benefit, in overall survival and radiological benefit. However, a potential prognostic relevance of DDR cannot be fully excluded. Differences in partial response rates were less marked. Unfortunately, neither of these reached statistical significance due to limited power but may offer promising therapeutic avenues for the future [30]. Identification of mutations in DDR genes could also help identifying patients who may benefit from other therapeutics targeting the DDR pathway such as poly ADP ribose polymerase (PARP) inhibitors or agents targeting Ataxia-Telangiesctasia mutated (ATM) or Ataxia telangiectasia and Rad3 related (ATR) [31]. However, whether genomic findings (mutations in DDR genes) derived on phenotypic changes (such as mismatch repair deficiency within others) was not explored in the present study and should be further evaluated to better understand the clinical significance of DDR-related gene mutations.

In clinical practice, the further development of precision medicine in BTC is challenging. First, most patients currently access molecular profiling through a clinical trial, which reflects the difficulties, not only in the United Kingdom but also in many European countries, of securing re-imbursement for molecular profiling for patients with BTC. Molecular profiling is likely to become standard of care for BTC and institutions should seek to address this need. Secondly, most patients approached were patients with iCCA; while this is probably linked to the fact that molecular profiling was mainly accessible through clinical trials targeting patients with cholangiocarcinoma only, it is vital that patients with non-iCCA BTC are also included as this field advances.

Limitations of this study are mainly related to the limited sample size and heterogeneity within molecular profiling panels employed. Molecular profiling was mainly access by recruitment into ongoing clinical trials, this being the reason why our population was biased towards iCCA (as we had recruiting studies focused on iCCA open at the time of these samples being analysed). In addition, this made our molecular profiling not consistent for all patients; thus, why all consecutive patients seen in our outpatient department could not be profiled, not allowing us to report a percentage of all patients with BTC who underwent molecular profiling. Limited sample size was of especial relevance at time of assessing concordance for matching samples and also when analysing the cohort of patients with ctDNA tested prior to the initiation of palliative therapy. Not all the panels targeted the same genes and the number of genes targeted also varied; even though the main cancer genes are well represented in all three panels, the effect of different technologies could not be fully excluded. Follow-up for some of the patients was limited which impacted on PFS and OS, with subsequent reduction in power for survival analysis. The fact that ctDNA was in occasions acquired following systemic chemotherapy challenges the interpretation of MAF, subclonal selection cannot be excluded either. Future studies, especially the ones focused on DDR, should also explore whether or not genomic changes do derive of phenotypic deficiencies or not, which was not addressed I the present series. 

In summary, these findings highlight that there is insufficient tumour content in archival tissue specimens in approximately 25% of patients with advanced BTC. ctDNA-based approaches may help to overcome this obstacle, with a more patient-friendly method to access molecular profiling for targeted therapy and tailored chemotherapy in advanced BTC. ctDNA identification was possible despite ongoing chemotherapy and there was high concordance with tissue, especially when focusing on the main genes of interest, such as IDH1 and FGFR2.

## Figures and Tables

**Table 1:** Summary of baseline patient characteristics.

Variable		All Patients (*n* = 104)		ctDNA Prior to Treatment Cohort (*n* = 12)	
		*n*	%	*n*	%
**Gender**	Female	52	50.0	10	83.3
	Male	52	50.0	2	16.7
**Age (years)**	Median (range)	62.5 (18.6–83.5)		67.4 (52.8–80.6)	
**Ethnic group**	British	87	91.6	9	75.0
	Other	8	8.4	3	25.0
**Primary tumour**	iCCA	71	68.2	8	66.7
	eCCA	24	23.1	1	8.3
	GBC	3	2.9	1	8.3
	Amp	6	5.8	2	16.7
**Stage**	Advanced (metastatic)	104	100	12	100
**Received palliative therapy**	Yes	98	94.2	12	100
	No	6	5.8	0	0.0
**Line of therapy (if palliative therapy)**	First-line	94	96.0	5	41.7
	Second-line	2	2.0	5	41.7
	Third-line	2	2.0	2	16.6
**Which palliative therapy (if palliative therapy)**	Cisplatin-gemcitabine	90	92.0	8	66.7
	FOLFIRINOX	2	2.0	0	0.0
	FOLFOX	2	2.0	3	25.0
	Cisplatin + NUC1031	2	2.0	0	0.0
	Gemcitabine	1	1.0	1	8.3
	SIRT	1	1.0	0	0.0
**Follow-up (months)**	Median (range)	12.5 (1.0–50.9)		8.4 (5.1–11.3)	
**Progression**	Yes	85	86.7	19	83.3
**PFS (months)**	Median (95% CI)	8.2 (6.9–9.0)		4.6 (2.4–8.7)	
**Died**	Yes	74	71.1	7	58.3
**OS from sample (months)**	Median (95% CI)	14.8 (12.3–20.3)		7.7 (5.9-not reached)	
**OS from palliative therapy initiation (months)**	Median (95% CI)	16.4 (13.3–19.7)		7.4 (4.6-not reached)	

*n*: number; %: percentage; ctDNA: circulating tumour DNA; DNA: Deoxyribonucleic acid; iCCA: intrahepatic cholangiocarcinoma; eCCA: extrahepatic cholangiocarcinoma; GBC: gallbladder cancer; Amp: ampulla of Vater carcinoma; FOLFIRINOX: 5-fluorouracil, irinotecan and oxaliplatin; FOLFOX: 5-fluorouracil and oxaliplatin; SIRT: Selective internal radiation therapy; CI: confidence interval; PFS: progression-free survival; and OS: overall survival.

**Table 2:** Molecular profiling-derived findings summary.

		All Samples (*n* = 112)	Tissue Sample (*n* = 90)	ctDNA (*n* = 22)	*p*-Value (Blood vs. ctDNA)
Pathological molecular findings	Yes	99; 88.4%	80; 88.9%	19; 86.4%	0.740
Targetable findings	Yes	45; 40.2% ^; 45.5% *	39; 43.3% ^; 48.6% *;	6; 27.3% ^; 31.6% *;	0.177
Presence of variants of unknown significance	Yes	76; 67.9%	60; 66.7%	16; 72.7%	0.585
Max MAF reported	Median (range)	n/a	n/a	0.89 (0.21–76.4)	n/a
TMB (Mut/Mb)	Median (range)	n/a	2 (0–6)	n/a	n/a

*n*: number; %: percentage; ctDNA: circulating tumour DNA; DNA: Deoxyribonucleic acid; mut: mutation; Mb: megabase; TMB: tumour mutational burden; MAF: mutant allele frequency; Max: maximum; n/a: not applicable; ^: percentage calculated out of the samples successfully analysed; and *: percentage calculated out of the samples with pathological molecular findings.

**Table 3:** Summary of pathological molecular findings by sample type.

		All Samples (*n* = 112)		Tissue Sample (*n* = 90)		ctDNA (*n* = 22)		ctDNA Prior to Palliative Therapy (*n* = 12)		Individual Patients (*n* = 89)		Targetable
		n	%	n	%	n	%	n	%	n	%	
ALK	Fusion	1	0.9	0	0.0	1	4.5	1	8.3	1	1.1	*
APC	Mutation	1	0.9	1	1.1	0	0.0	0	0.0	1	1.1	
ARID1A	Mutation	6	5.4	6	6.7	0	0.0	0	0.0	6	6.7	
ATM	Mutation	5	4.5	3	3.3	2	9.1	1	8.3	4	4.5	
ATRX	Mutation	2	1.8	2	2.2	0	0.0	0	0.0	2	2.2	
BAP1	Mutation	8	7.1	8	8.9	0	0.0	0	0.0	8	9.0	
BRAF	Mutation	1	0.9	1	1.1	0	0.0	0	0.0	1	1.1	*
BRCA1	Mutation	1	0.9	1	1.1	0	0.0	0	0.0	1	1.1	
CCDN1	Amplification	1	0.9	1	1.1	0	0.0	0	0.0	1	1.1	
CDK4	Amplification	2	1.8	2	2.2	0	0.0	0	0.0	2	2.2	
CDKN2A	Mutation	11	9.8	11	12.2	0	0.0	0	0.0	11	12.4	
CHECK2	Mutation	1	0.9	0	0.0	1	4.5	0	0.0	1	1.1	
CTNNB1	Mutation	1	0.9	0	0.0	1	4.5	0	0.0	1	1.1	
ERRB2	Amplification	2	1.8	2	2.2	0	0.0	0	0.0	2	2.2	*
ERRB3	Amplification	3	2.7	3	3.3	0	0.0	0	0.0	3	3.4	*
FGFR2	Mutation	7	6.3	6	6.7	1	4.5	1	8.3	5	5.6	*
FGFR2	Fusion	12	10.7	8	8.9	4	18.2	1	8.3	9	10.1	*
GNAS	Mutation	1	0.9	0	0.0	1	4.5	1	8.3	1	1.1	
IDH1	Mutation	21	18.8	17	18.9	4	18.2	2	16.7	17	19.1	*
IDH2	Mutation	6	5.4	6	6.7	0	0.0	0	0.0	5	5.6	*
KRAS	Mutation	28	25.0	27	30.0	1	4.5	0	0.0	24	27.0	
MDM2	Amplification	6	5.4	6	6.7	0	0.0	0	0.0	6	6.7	
MET	Mutation	1	0.9	1	1.1	0	0.0	0	0.0	1	1.1	
MET	Amplification	1	0.9	1	1.1	0	0.0	0	0.0	1	1.1	
MSH6	Mutation	1	0.9	1	1.1	0	0.0	0	0.0	1	1.1	
NF1	Mutation	4	3.6	1	1.1	3	13.6	2	16.7	4	4.5	
NRAS	Mutation	1	0.9	1	1.1	0	0.0	0	0.0	1	1.1	
PI3KCA	Mutation	4	3.6	3	3.3	1	4.5	0	0.0	3	3.4	
POLD1	Mutation	1	0.9	1	1.1	0	0.0	0	0.0	1	1.1	
PTEN	Mutation	5	4.5	4	4.4	1	4.5	0	0.0	4	4.5	
RB1	Mutation	1	0.9	1	1.1	0	0.0	0	0.0	1	1.1	
RNF43	Mutation	1	0.9	1	1.1	0	0.0	0	0.0	1	1.1	*
SMAD4	Mutation	6	5.4	6	6.7	0	0.0	0	0.0	6	6.7	
TP53	Mutation	22	19.6	14	15.6	8	36.4	7	58.3	22	24.7	
Other	Mutation	36	32.1	33	36.7	3	13.6	1	8.3	36	40.4	
Other	Amplification	16	14.3	16	17.8	0	0.0	0	0.0	16	18.0	

*n*: number; %: percentage; ctDNA: circulating tumour DNA; DNA: Deoxyribonucleic acid; and mut: mutation. * highlight potentially “targetable” alterations in biliary tract cancer. Percentages are calculated for successful-analysed samples/patients.

## Supplementary Materials

The following are available online at https://www.mdpi.com/2077-0383/9/9/2854/s1, Table S1: Baseline characteristics by sample type, Table S2: Pathological molecular findings by primary tumour, Table S3: Pathological molecular findings for individual patients diagnosed with intrahepatic cholangiocarcinoma, Table S4: Pathological molecular findings for individual patients diagnosed with extrahepatic cholangiocarcinoma, Table S5: Pathological molecular findings for individual patient diagnosed with gallbladder adenocarcinoma, Table S6: Pathological molecular findings for individual patients diagnosed with ampullary carcinoma, Table S7: Paired tissue samples, Table S8: ctDNA prior to treatment, Table S9: Baseline characteristics by presence/absence of DDR gene alterations.
