# Supplementary material for: Molecular Profiling in Daily Clinical Practice: Practicalities in Advanced Cholangiocarcinoma and Other Biliary Tract Cancers
Source: J Clin Med. 2020 Sep 3;9(9):2854. doi: 10.3390/jcm9092854 (PMC7563385; doi:10.3390/jcm9092854)
Supplement: Supplementary file 1 [file jcm-09-02854-s001.zip › jcm-914593 supplementary materials/jcm-914593 supplementary material.docx]

**Supplementary material**

**Table S1.** Baseline characteristics by sample type.

|  | | **Tissue sample (*n* = 123)** | | **ctDNA (*n* = 26)** | | **p-value (tissue vs ctDNA)** |
| --- | --- | --- | --- | --- | --- | --- |
|  |  | ***n*** | **%** | ***n*** | **%** |  |
| **Gender** | Female | 58 | 47.2 | 18 | 69.2 | 0.041 |
|  | Male | 65 | 52.6 | 8 | 30.8 |  |
| **Age (years)** | Median (range) | 61.6 (18.6-83.5) | | 67.4 (31.2-80.6) | | 0.027 |
| **Ethnic group** | British | 105 | 92.1 | 20 | 83.3 | 0.409 |
|  | Other | 18 | 7.9 | 6 | 16.7 |  |
| **Primary tumour** | iCCA | 87 | 70.7 | 15 | 57.7 | 0.038 |
|  | eCCA | 31 | 25.2 | 6 | 23.0 |  |
|  | GBC | 2 | 1.6 | 1 | 3.9 |  |
|  | Amp | 3 | 2.5 | 4 | 15.4 |  |
| **Stage** | Advanced (metastatic) | 123 | 100.0 | 26 | 100.0 | *n*/a |
| **Received palliative therapy** | Yes | 117 | 95.1 | 25 | 96.1 | 1.000 |
|  | No | 6 | 4.9 | 1 | 3.9 |  |
| **Line of therapy (if palliative therapy)** | First-line | 114 | 97.4 | 13 | 52.2 | <0.001 |
|  | Second-line | 3 | 2.6 | 9 | 36.0 |  |
|  | Third-line | 0 | 0.0 | 3 | 12.0 |  |
| **Which palliative therapy (if palliative therapy)** | Cisplatin-gemcitabine | 120 | 97.4 | 14 | 53.8 | <0.001 |
|  | FOLFIRINOX | 2 | 1.7 | 1 | 4.0 |  |
|  | FOLFOX | 1 | 0.9 | 5 | 20.0 |  |
|  | Cisplatin + NUC1031 | 0 | 0.0 | 2 | 8.0 |  |
|  | Gemcitabine | 0 | 0.0 | 1 | 4.0 |  |
|  | SIRT | 0 | 0.0 | 1 | 4.0 |  |
|  | FGFR2 inhibitor | 0 | 0.0 | 2 | 8.0 |  |
| **Follow-up (months)** | Median (range) | 14.8 (0.7-65.7) | | 7.4 (1.4-11.3) | | <0.001 |

Ttest, Chi-Square of Fisher’s exact p-values are provided as appropriate. *n*: number; %: percentage; ctDNA: circulating tumour DNA; DNA: Deoxyribonucleic acid; iCCA: intrahepatic cholangiocarcinoma; eCCA: extrahepatic cholangiocarcinoma; GBC: gallbladder cancer; Amp: ampulla of Vater carcinoma; *n*/a: not applicable; FOLFIRINOX: 5-fluorouracil, irinotecan and oxaliplatin; FOLFOX: 5-fluorouracil and oxaliplatin; SIRT: Selective internal radiation therapy; CI: confidence interval; FGFR: fibroblast growth factor receptor; PFS: progression-free survival; OS: overall survival.

**Table S2.** Pathological molecular findings by primary tumour.

|  | **iCCA** | | **eCCA** | | **AMP** | | **Targetable** |
| --- | --- | --- | --- | --- | --- | --- | --- |
|  | Frequency per sample (%) (*n* = 76) | Frequency per patient (%) (*n* = 62) | Frequency per sample (%) (*n* = 27) | Frequency per patient (%) (*n* = 20) | Frequency per sample (%) (*n* = 7) | Frequency per patient (%) (*n* = 6) |  |
| ALK | 0.0 | 0.0 | 0.0 | 0.0 | 0.0 | 0.0 | * |
| APC | 0.0 | 0.0 | 0.0 | 0.0 | 14.3 | 16.7 |  |
| ARID1A | 6.6 | 8.1 | 3.7 | 5.0 | 0.0 | 0.0 |  |
| ATM | 3.9 | 4.8 | 0.0 | 0.0 | 28.6 | 16.7 |  |
| ATRX | 2.6 | 3.2 | 0.0 | 0.0 | 0.0 | 0.0 |  |
| BAP1 | 9.2 | 11.3 | 0.0 | 0.0 | 0.0 | 0.0 |  |
| BRAF | 1.3 | 1.6 | 0.0 | 0.0 | 0.0 | 0.0 | * |
| BRCA1 | 1.3 | 1.6 | 0.0 | 0.0 | 0.0 | 0.0 |  |
| CCND1 | 1.3 | 1.6 | 0.0 | 0.0 | 0.0 | 0.0 |  |
| CDK4 | 1.3 | 1.6 | 0.0 | 0.0 | 14.3 | 16.7 |  |
| CDKN2A | 13.2 | 16.1 | 0.0 | 0.0 | 14.3 | 16.7 |  |
| CHEK2 | 0.0 | 0.0 | 3.7 | 5.0 | 0.0 | 0.0 |  |
| CTNNB1 | 0.0 | 0.0 | 3.7 | 5.0 | 0.0 | 0.0 |  |
| ERRB2 | 1.3 | 1.6 | 3.7 | 5.0 | 0.0 | 0.0 | * |
| ERRB3 | 2.6 | 3.2 | 0.0 | 0.0 | 14.3 | 16.7 | * |
| FGFR2 | 22.4 | 21.0 | 3.7 | 5.0 | 0.0 | 0.0 | * |
| GNAS | 1.3 | 1.6 | 0.0 | 0.0 | 0.0 | 0.0 |  |
| IDH1 | 25.0 | 25.8 | 7.4 | 5.0 | 0.0 | 0.0 | * |
| IDH2 | 7.9 | 9.7 | 0.0 | 0.0 | 0.0 | 0.0 | * |
| KRAS | 18.4 | 21.0 | 48.1 | 55.0 | 14.3 | 16.7 |  |
| MDM2 | 1.3 | 1.6 | 11.1 | 15.0 | 28.6 | 33.3 |  |
| MET | 2.6 | 3.2 | 0.0 | 0.0 | 0.0 | 0.0 |  |
| MSH6 | 0.0 | 0.0 | 3.7 | 5.0 | 0.0 | 0.0 |  |
| NF1 | 5.3 | 6.5 | 0.0 | 0.0 | 0.0 | 0.0 |  |
| NRAS | 1.3 | 1.6 | 0.0 | 0.0 | 0.0 | 0.0 |  |
| PIK3CA | 5.3 | 4.8 | 0.0 | 0.0 | 0.0 | 0.0 |  |
| POLD1 | 0.0 | 0.0 | 3.7 | 5.0 | 0.0 | 0.0 |  |
| PTEN | 3.9 | 3.2 | 0.0 | 0.0 | 28.6 | 33.3 |  |
| RB1 | 1.3 | 1.6 | 0.0 | 0.0 | 0.0 | 0.0 |  |
| RNF43 | 1.3 | 1.6 | 3.7 | 5.0 | 0.0 | 0.0 | * |
| SMAD4 | 5.3 | 6.5 | 3.7 | 5.0 | 14.3 | 16.7 |  |
| TP53 | 13.2 | 16.1 | 29.6 | 40.0 | 42.9 | 50.0 |  |
| Other mut | 36.8 | 45.2 | 22.2 | 30.0 | 28.6 | 33.3 |  |
| Other ampl | 14.5 | 17.7 | 14.8 | 20.0 | 14.3 | 16.7 |  |

Only 1 patient with gallbladder cancer was analysed (**Table S5**), thus not represented in this summary table. Data for individual patients with iCCA, eCCA and Amp are provided in **Table S3**, **Table S4** and **Table S6**, respectively.*n*: number; %: percentage; ctDNA: circulating tumour DNA; DNA: Deoxyribonucleic acid; mut: mutation; iCCA: intrahepatic cholangiocarcinoma; eCCA: extrahepatic cholangiocarcinoma; Amp: ampulla of Vater carcinoma. * highlight potentially “targetable” alterations in biliary tract cancer.

**Table S3.** Pathological molecular findings for individual patients diagnosed with intrahepatic cholangiocarcinoma.

iCCA: intrahepatic cholangiocarcinoma; FM: Foundation Medicine®, refers to FoundationOne CDx® or FoundationOne Liquid® panel; Onc: Oncomine® panel; B: blood (ctDNA); T: tumour tissue; %: percentage; Pt: patients ID; Mut: mutations; Mb: megabase; TMB: tumour mutational burden. Red box: mutation; blue box: amplification; green box: fusion.

**Table S4.** Pathological molecular findings for individual patients diagnosed with extrahepatic cholangiocarcinoma.

| **Patient** | **Pt 6** | **Pt 6** | **Pt 7** | **Pt 14** | **Pt 15** | **Pt 15** | **Pt 17** | **Pt 18** | **Pt 19** | **Pt 19** | **Pt 21** | **Pt 22** | **Pt 23** | **Pt 23** | **Pt 24** | **Pt 24** | **Pt 25** | **Pt 29** | **Pt 29** | **Pt 36** | **Pt 41** | **Pt 41** | **Pt 46** | **Pt 47** | **Pt 58** | **Pt 64** | **Pt 77** | **Frequency per sample (%)** | **Frequency per patient (%)** |
| --- | --- | --- | --- | --- | --- | --- | --- | --- | --- | --- | --- | --- | --- | --- | --- | --- | --- | --- | --- | --- | --- | --- | --- | --- | --- | --- | --- | --- | --- |
| **Primary** | **eCCA** | **eCCA** | **eCCA** | **eCCA** | **eCCA** | **eCCA** | **eCCA** | **eCCA** | **eCCA** | **eCCA** | **eCCA** | **eCCA** | **eCCA** | **eCCA** | **eCCA** | **eCCA** | **eCCA** | **eCCA** | **eCCA** | **eCCA** | **eCCA** | **eCCA** | **eCCA** | **eCCA** | **eCCA** | **eCCA** | **eCCA** |  |  |
| **Test** | **FM** | **Onc** | **FM** | **Onc** | **FM** | **Onc** | **FM** | **Onc** | **FM** | **Onc** | **Onc** | **Onc** | **FM** | **Onc** | **FM** | **Onc** | **FM** | **FM** | **Onc** | **Onc** | **FM** | **Onc** | **Onc** | **Onc** | **FM** | **FM** | **FM** |  |  |
| **Fusion analyis** | yes | yes | yes | failed | yes | yes | yes | failed | yes | yes | failed | yes | yes | yes | yes | yes | yes | yes | yes | yes | yes | yes | yes | yes | yes | yes | yes |  |  |
| **Sample** | **T** | **T** | **T** | **T** | **T** | **T** | **T** | **T** | **T** | **T** | **T** | **T** | **T** | **T** | **T** | **T** | **B** | **T** | **T** | **T** | **T** | **T** | **T** | **T** | **B** | **B** | **B** |  |  |
| **ALK** |  |  |  |  |  |  |  |  |  |  |  |  |  |  |  |  |  |  |  |  |  |  |  |  |  |  |  | 0.0 | 0.0 |
| **APC** |  |  |  |  |  |  |  |  |  |  |  |  |  |  |  |  |  |  |  |  |  |  |  |  |  |  |  | 0.0 | 0.0 |
| **ARID1A** |  |  |  |  |  |  |  |  |  |  |  |  |  |  |  |  |  |  |  |  |  |  |  |  |  |  |  | 3.7 | 5.0 |
| **ATM** |  |  |  |  |  |  |  |  |  |  |  |  |  |  |  |  |  |  |  |  |  |  |  |  |  |  |  | 0.0 | 0.0 |
| **ATRX** |  |  |  |  |  |  |  |  |  |  |  |  |  |  |  |  |  |  |  |  |  |  |  |  |  |  |  | 0.0 | 0.0 |
| **BAP1** |  |  |  |  |  |  |  |  |  |  |  |  |  |  |  |  |  |  |  |  |  |  |  |  |  |  |  | 0.0 | 0.0 |
| **BRAF** |  |  |  |  |  |  |  |  |  |  |  |  |  |  |  |  |  |  |  |  |  |  |  |  |  |  |  | 0.0 | 0.0 |
| **BRCA1** |  |  |  |  |  |  |  |  |  |  |  |  |  |  |  |  |  |  |  |  |  |  |  |  |  |  |  | 0.0 | 0.0 |
| **CCND1** |  |  |  |  |  |  |  |  |  |  |  |  |  |  |  |  |  |  |  |  |  |  |  |  |  |  |  | 0.0 | 0.0 |
| **CDK4** |  |  |  |  |  |  |  |  |  |  |  |  |  |  |  |  |  |  |  |  |  |  |  |  |  |  |  | 0.0 | 0.0 |
| **CDKN2A** |  |  |  |  |  |  |  |  |  |  |  |  |  |  |  |  |  |  |  |  |  |  |  |  |  |  |  | 0.0 | 0.0 |
| **CHEK2** |  |  |  |  |  |  |  |  |  |  |  |  |  |  |  |  |  |  |  |  |  |  |  |  |  |  |  | 3.7 | 5.0 |
| **CTNNB1** |  |  |  |  |  |  |  |  |  |  |  |  |  |  |  |  |  |  |  |  |  |  |  |  |  |  |  | 3.7 | 5.0 |
| **ERRB2** |  |  |  |  |  |  |  |  |  |  |  |  |  |  |  |  |  |  |  |  |  |  |  |  |  |  |  | 3.7 | 5.0 |
| **ERRB3** |  |  |  |  |  |  |  |  |  |  |  |  |  |  |  |  |  |  |  |  |  |  |  |  |  |  |  | 0.0 | 0.0 |
| **FGFR2** |  |  |  |  |  |  |  |  |  |  |  |  |  |  |  |  |  |  |  |  |  |  |  |  |  |  |  | 3.7 | 5.0 |
| **GNAS** |  |  |  |  |  |  |  |  |  |  |  |  |  |  |  |  |  |  |  |  |  |  |  |  |  |  |  | 0.0 | 0.0 |
| **IDH1** |  |  |  |  |  |  |  |  |  |  |  |  |  |  |  |  |  |  |  |  |  |  |  |  |  |  |  | 7.4 | 5.0 |
| **IDH2** |  |  |  |  |  |  |  |  |  |  |  |  |  |  |  |  |  |  |  |  |  |  |  |  |  |  |  | 0.0 | 0.0 |
| **KRAS** |  |  |  |  |  |  |  |  |  |  |  |  |  |  |  |  |  |  |  |  |  |  |  |  |  |  |  | 48.1 | 55.0 |
| **MDM2** |  |  |  |  |  |  |  |  |  |  |  |  |  |  |  |  |  |  |  |  |  |  |  |  |  |  |  | 11.1 | 15.0 |
| **MET** |  |  |  |  |  |  |  |  |  |  |  |  |  |  |  |  |  |  |  |  |  |  |  |  |  |  |  | 0.0 | 0.0 |
| **MSH6** |  |  |  |  |  |  |  |  |  |  |  |  |  |  |  |  |  |  |  |  |  |  |  |  |  |  |  | 3.7 | 5.0 |
| **NF1** |  |  |  |  |  |  |  |  |  |  |  |  |  |  |  |  |  |  |  |  |  |  |  |  |  |  |  | 0.0 | 0.0 |
| **NRAS** |  |  |  |  |  |  |  |  |  |  |  |  |  |  |  |  |  |  |  |  |  |  |  |  |  |  |  | 0.0 | 0.0 |
| **PIK3CA** |  |  |  |  |  |  |  |  |  |  |  |  |  |  |  |  |  |  |  |  |  |  |  |  |  |  |  | 0.0 | 0.0 |
| **POLD1** |  |  |  |  |  |  |  |  |  |  |  |  |  |  |  |  |  |  |  |  |  |  |  |  |  |  |  | 3.7 | 5.0 |
| **PTEN** |  |  |  |  |  |  |  |  |  |  |  |  |  |  |  |  |  |  |  |  |  |  |  |  |  |  |  | 0.0 | 0.0 |
| **RB1** |  |  |  |  |  |  |  |  |  |  |  |  |  |  |  |  |  |  |  |  |  |  |  |  |  |  |  | 0.0 | 0.0 |
| **RNF43** |  |  |  |  |  |  |  |  |  |  |  |  |  |  |  |  |  |  |  |  |  |  |  |  |  |  |  | 3.7 | 5.0 |
| **SMAD4** |  |  |  |  |  |  |  |  |  |  |  |  |  |  |  |  |  |  |  |  |  |  |  |  |  |  |  | 3.7 | 5.0 |
| **TP53** |  |  |  |  |  |  |  |  |  |  |  |  |  |  |  |  |  |  |  |  |  |  |  |  |  |  |  | 29.6 | 40.0 |
| **Other mut** |  |  |  |  |  |  |  |  |  |  |  |  |  |  |  |  |  |  |  |  |  |  |  |  |  |  |  | 22.2 | 30.0 |
| **Other ampl** |  |  |  |  |  |  |  |  |  |  |  |  |  |  |  |  |  |  |  |  |  |  |  |  |  |  |  | 14.8 | 20.0 |
| **TMB (Mut/Mb)** |  |  |  |  |  |  | 2 |  |  |  |  |  |  |  |  |  |  |  |  |  |  |  |  |  |  |  |  |  |  |

eCCA: extrahepatic cholangiocarcinoma; FM: Foundation Medicine®, refers to FoundationOne CDx® or FoundationOne Liquid® panel; Onc: Oncomine® panel; B: blood (ctDNA); T: tumour tissue; %: percentage; Pt: patients ID; Mut: mutations; Mb: megabase; TMB: tumour mutational burden. Red box: mutation; blue box: amplification; green box: fusion.

**Table S5.** Pathological molecular findings for individual patient diagnosed with gallbladder adenocarcinoma.

| Patient | Pt 70 |
| --- | --- |
| Primary | GBC |
| Test | FM |
| Fusion analyis | yes |
| Sample | B |
| ALK |  |
| APC |  |
| ARID1A |  |
| ATM |  |
| ATRX |  |
| BAP1 |  |
| BRAF |  |
| BRCA1 |  |
| CCND1 |  |
| CDK4 |  |
| CDKN2A |  |
| CHEK2 |  |
| CTNNB1 |  |
| ERRB2 |  |
| ERRB3 |  |
| FGFR2 |  |
| GNAS |  |
| IDH1 |  |
| IDH2 |  |
| KRAS |  |
| MDM2 |  |
| MET |  |
| MSH6 |  |
| NF1 |  |
| NRAS |  |
| PIK3CA |  |
| POLD1 |  |
| PTEN |  |
| RB1 |  |
| RNF43 |  |
| SMAD4 |  |
| TP53 |  |
| Other mut |  |
| Other ampl |  |
| TMB (Mut/Mb) |  |

GBC: gallbaldder adenocarcinoma; FM: Foundation Medicine®, refers to FoundationOne Liquid® panel; B: blood (ctDNA); %: percentage; Pt: patients ID; Mut: mutations; Mb: megabase; TMB: tumour mutational burden. Red box: mutation; blue box: amplification; green box: fusion.

**Table S6** Pathological molecular findings for individual patients diagnosed with ampullary carcinoma.

| **Patient** | **Pt 1** | **Pt 8** | **Pt 10** | **Pt 12** | **Pt 35** | **Pt 35** | **Pt 75** | **Frequency per sample (%)** | **Frequency per patient (%)** |
| --- | --- | --- | --- | --- | --- | --- | --- | --- | --- |
| **Primary** | **AMP** | **AMP** | **AMP** | **AMP** | **AMP** | **AMP** | **AMP** |  |  |
| **Test** | **FM** | **FM** | **FM** | **FM** | **FM** | **FM** | **FM** |  |  |
| **Fusion analyis** | yes | yes | yes | yes | yes | yes | yes |  |  |
| **Sample** | **B** | **T** | **T** | **B** | **T** | **B** | **B** |  |  |
| **ALK** |  |  |  |  |  |  |  | 0.0 | 0.0 |
| **APC** |  |  |  |  |  |  |  | 14.3 | 16.7 |
| **ARID1A** |  |  |  |  |  |  |  | 0.0 | 0.0 |
| **ATM** |  |  |  |  |  |  |  | 28.6 | 16.7 |
| **ATRX** |  |  |  |  |  |  |  | 0.0 | 0.0 |
| **BAP1** |  |  |  |  |  |  |  | 0.0 | 0.0 |
| **BRAF** |  |  |  |  |  |  |  | 0.0 | 0.0 |
| **BRCA1** |  |  |  |  |  |  |  | 0.0 | 0.0 |
| **CCND1** |  |  |  |  |  |  |  | 0.0 | 0.0 |
| **CDK4** |  |  |  |  |  |  |  | 14.3 | 16.7 |
| **CDKN2A** |  |  |  |  |  |  |  | 14.3 | 16.7 |
| **CHEK2** |  |  |  |  |  |  |  | 0.0 | 0.0 |
| **CTNNB1** |  |  |  |  |  |  |  | 0.0 | 0.0 |
| **ERRB2** |  |  |  |  |  |  |  | 0.0 | 0.0 |
| **ERRB3** |  |  |  |  |  |  |  | 14.3 | 16.7 |
| **FGFR2** |  |  |  |  |  |  |  | 0.0 | 0.0 |
| **GNAS** |  |  |  |  |  |  |  | 0.0 | 0.0 |
| **IDH1** |  |  |  |  |  |  |  | 0.0 | 0.0 |
| **IDH2** |  |  |  |  |  |  |  | 0.0 | 0.0 |
| **KRAS** |  |  |  |  |  |  |  | 14.3 | 0.0 |
| **MDM2** |  |  |  |  |  |  |  | 28.6 | 33.3 |
| **MET** |  |  |  |  |  |  |  | 0.0 | 0.0 |
| **MSH6** |  |  |  |  |  |  |  | 0.0 | 0.0 |
| **NF1** |  |  |  |  |  |  |  | 0.0 | 0.0 |
| **NRAS** |  |  |  |  |  |  |  | 0.0 | 0.0 |
| **PIK3CA** |  |  |  |  |  |  |  | 0.0 | 0.0 |
| **POLD1** |  |  |  |  |  |  |  | 0.0 | 0.0 |
| **PTEN** |  |  |  |  |  |  |  | 28.6 | 33.3 |
| **RB1** |  |  |  |  |  |  |  | 0.0 | 0.0 |
| **RNF43** |  |  |  |  |  |  |  | 0.0 | 0.0 |
| **SMAD4** |  |  |  |  |  |  |  | 14.3 | 16.7 |
| **TP53** |  |  |  |  |  |  |  | 42.9 | 50.0 |
| **Other mutation** |  |  |  |  |  |  |  | 28.6 | 33.3 |
| **Other amplification** |  |  |  |  |  |  |  | 14.3 | 16.7 |
| **TMB (Mut/Mb)** |  | 1 | 6 |  | 0 |  |  |  |  |

AMP: ampullary adenocarcinoma; FM: Foundation Medicine®, refers to FoundationOne CDx® or FoundationOne Liquid® panel; Onc: Oncomine® panel; B: blood (ctDNA); T: tumour tissue; %: percentage; Pt: patients ID; Mut: mutations; Mb: megabase; TMB: tumour mutational burden. Red box: mutation; blue box: amplification; green box: fusion.

**Table S7.** Paired tissue samples.

iCCA: intrahepatic cholangiocarcinoma; eCCA: extrahepatic cholangiocarcinoma; GBC: gallbladder cancer; Amp: ampulla of Vater carcinoma; FM: Foundation Medicine®, refers to FoundationOne CDx® or FoundationOne Liquid® panel; Onc: Oncomine® panel; B: blood (ctDNA); T: tumour tissue; %: percentage; Pt: patients. ID; Mut: mutations; Mb: megabase; TMB: tumour mutational burden; amplif: amplifications. Red box: mutation; blue box: amplification; green box: fusion; yellow box: targetable alteration; orange: frequent but non-targetable alteration. *: * fusion analysis failed so 1/1 instead of 1/2.

**Table S8.** ctDNA prior to treatment.

| **Patient** | **Pt 3** | **Pt 3** | **Pt 3** | **Pt 9** | **Pt 9** | **Pt 30** | **Pt 30** | **Pt 33** | **Pt 33** | **Pt 35** | **Pt 35** | **Pt 56** | **Pt 56** | **Pt 60** | **Pt 60** | **Pt 79** | **Pt 79** | **Pt 83** | **Pt 83** | **Agreement** |
| --- | --- | --- | --- | --- | --- | --- | --- | --- | --- | --- | --- | --- | --- | --- | --- | --- | --- | --- | --- | --- |
| **Primary** | **iCCA** | **iCCA** | **iCCA** | **iCCA** | **iCCA** | **iCCA** | **iCCA** | **iCCA** | **iCCA** | **AMP** | **AMP** | **iCCA** | **iCCA** | **iCCA** | **iCCA** | **iCCA** | **iCCA** | **iCCA** | **iCCA** |  |
| **Test** | **FM** | **Onc** | **FM** | **FM** | **FM** | **FM** | **FM** | **FM** | **FM** | **FM** | **FM** | **FM** | **FM** | **FM** | **FM** | **FM** | **FM** | **FM** | **FM** |  |
| **Fusion analyis** | yes | yes | yes | yes | yes | yes | yes | yes | yes | yes | yes | yes | yes | yes | yes | yes | yes | yes | yes |  |
| **Sample** | **T** | **T** | **B** | **T** | **B** | **T** | **B** | **T** | **B** | **T** | **B** | **T** | **B** | **T** | **B** | **T** | **B** | **T** | **B** |  |
| **ALK** |  |  |  |  |  |  |  |  |  |  |  |  |  |  |  |  |  |  |  | - |
| **APC** |  |  |  |  |  |  |  |  |  |  |  |  |  |  |  |  |  |  |  | - |
| **ARID1A** |  |  |  |  |  |  |  |  |  |  |  |  |  |  |  |  |  |  |  | 0/1 |
| **ATM** |  |  |  |  |  |  |  |  |  |  |  |  |  |  |  |  |  |  |  | 1/1 |
| **ATRX** |  |  |  |  |  |  |  |  |  |  |  |  |  |  |  |  |  |  |  | 0/1 |
| **BAP1** |  |  |  |  |  |  |  |  |  |  |  |  |  |  |  |  |  |  |  | 0/2 |
| **BRAF** |  |  |  |  |  |  |  |  |  |  |  |  |  |  |  |  |  |  |  | - |
| **BRCA1** |  |  |  |  |  |  |  |  |  |  |  |  |  |  |  |  |  |  |  | - |
| **CCND1** |  |  |  |  |  |  |  |  |  |  |  |  |  |  |  |  |  |  |  | - |
| **CDK4** |  |  |  |  |  |  |  |  |  |  |  |  |  |  |  |  |  |  |  | 0/2 |
| **CDKN2A** |  |  |  |  |  |  |  |  |  |  |  |  |  |  |  |  |  |  |  | 0/3 |
| **CHEK2** |  |  |  |  |  |  |  |  |  |  |  |  |  |  |  |  |  |  |  | - |
| **CTNNB1** |  |  |  |  |  |  |  |  |  |  |  |  |  |  |  |  |  |  |  | - |
| **ERRB2** |  |  |  |  |  |  |  |  |  |  |  |  |  |  |  |  |  |  |  | - |
| **ERRB3** |  |  |  |  |  |  |  |  |  |  |  |  |  |  |  |  |  |  |  | 0/1 |
| **FGFR2** |  |  |  |  |  |  |  |  |  |  |  |  |  |  |  |  |  |  |  | 3/3 |
| **GNAS** |  |  |  |  |  |  |  |  |  |  |  |  |  |  |  |  |  |  |  | - |
| **IDH1** |  |  |  |  |  |  |  |  |  |  |  |  |  |  |  |  |  |  |  | 3/3 |
| **IDH2** |  |  |  |  |  |  |  |  |  |  |  |  |  |  |  |  |  |  |  | - |
| **KRAS** |  |  |  |  |  |  |  |  |  |  |  |  |  |  |  |  |  |  |  | - |
| **MDM2** |  |  |  |  |  |  |  |  |  |  |  |  |  |  |  |  |  |  |  | 0/2 |
| **MET** |  |  |  |  |  |  |  |  |  |  |  |  |  |  |  |  |  |  |  | 0/1 |
| **MSH6** |  |  |  |  |  |  |  |  |  |  |  |  |  |  |  |  |  |  |  | - |
| **NF1** |  |  |  |  |  |  |  |  |  |  |  |  |  |  |  |  |  |  |  | 0/2 |
| **NRAS** |  |  |  |  |  |  |  |  |  |  |  |  |  |  |  |  |  |  |  | - |
| **PIK3CA** |  |  |  |  |  |  |  |  |  |  |  |  |  |  |  |  |  |  |  | 1/1 |
| **POLD1** |  |  |  |  |  |  |  |  |  |  |  |  |  |  |  |  |  |  |  | - |
| **PTEN** |  |  |  |  |  |  |  |  |  |  |  |  |  |  |  |  |  |  |  | 1/3 |
| **RB1** |  |  |  |  |  |  |  |  |  |  |  |  |  |  |  |  |  |  |  | - |
| **RNF43** |  |  |  |  |  |  |  |  |  |  |  |  |  |  |  |  |  |  |  | - |
| **SMAD4** |  |  |  |  |  |  |  |  |  |  |  |  |  |  |  |  |  |  |  | 0/1 |
| **TP53** |  |  |  |  |  |  |  |  |  |  |  |  |  |  |  |  |  |  |  | 0/2 |
| **Other mut** |  |  |  |  |  |  |  |  |  |  |  |  |  |  |  |  |  |  |  | 0/6 |
| **Other ampl** |  |  |  |  |  |  |  |  |  |  |  |  |  |  |  |  |  |  |  | 0/3 |

iCCA: intrahepatic cholangiocarcinoma; eCCA: extrahepatic cholangiocarcinoma; GBC: gallbladder cancer; Amp: ampulla of Vater carcinoma; FM: Foundation Medicine®, refers to FoundationOne Liquid® panel; B: blood (ctDNA); %: percentage; Pt: patients ID; Mut: mutations; Mb: megabase; TMB: tumour mutational burden; amplif: amplifications. Red box: mutation; blue box: amplification; green box: fusion. Targetable findings: ALK, IDH1, FGFR2.

**Table S9.** Baseline characteristics by presence/absence of DDR gene alterations.

|  | | **Presence of DDR gene alterations (*n* = 15)** | | **Absence of DDR gene alterations (*n* = 74)** | | p-value (presence vs absence) |
| --- | --- | --- | --- | --- | --- | --- |
|  |  | ***n*** | **%** | ***n*** | **%** |  |
| **Which DDR gene mutated** | ATM | 4 | 26.6% | - | - | *n*/a |
|  | BAP1 | 7 | 46.6% | - | - |  |
|  | BRCA1 | 1 | 6.7% | - | - |  |
|  | CHEK2 | 1 | 6.7% | - | - |  |
|  | MSH6* | 1 | 6.7% | - | - |  |
|  | POLD1* |  |  | - | - |  |
|  | FANCA | 1 | 6.7% | - | - |  |
| **Gender** | Female | 8 | 53.3 | 37 | 50.0 | 0.814 |
|  | Male | 7 | 46.7 | 37 | 50.0 |  |
| **Age (years)** | Median (range) | 64.9 (30.5-83.5) | | 61.7 (25.8-80.7) | | 0.3198 |
| **Ethnic group** | British | 13 | 91.9 | 62 | 91.2 | 0.515 |
|  | Other | 2 | 8.1 | 12 | 8.8 |  |
| **Primary tumour** | iCCA | 12 | 80.0 | 50 | 67.6 | 0.827 |
|  | eCCA | 2 | 13.3 | 18 | 24.3 |  |
|  | GBC | 0 | 0.0 | 1 | 1.4 |  |
|  | Amp | 1 | 6.7 | 5 | 6.7 |  |
| **Stage** | Advanced (metastatic) | 15 | 100.0 | 74 | 100.0 | *n*/a |
| **Received palliative therapy** | Yes | 14 | 93.3 | 69 | 93.2 | 1.000 |
|  | No | 1 | 6.7 | 5 | 6.8 |  |
| **Line of therapy (if palliative therapy)** | First-line | 13 | 92.9 | 64 | 92.8 | 0.682 |
|  | Second-line | 1 | 7.1 | 3 | 4.3 |  |
|  | Third-line | 0 | 0.0 | 2 | 2.9 |  |
| **Which palliative therapy (if palliative therapy)** | Cisplatin-gemcitabine | 13 | 92.9 | 63 | 91.1 | 0.860 |
|  | FOLFIRINOX | 0 | 0.0 | 1 | 1.5 |  |
|  | FOLFOX | 1 | 7.1 | 3 | 4.4 |  |
|  | Gemcitabine | 0 | 0.0 | 1 | 1.5 |  |
|  | SIRT | 0 | 0.0 | 1 | 1.5 |  |
| **Follow-up (months)** | Median (range) | 12.3 (1.4-46.0) | | 11.3 (0.7-50.9) | | 0.4872 |
| **OS from sample (months) (all patients; *n* = 89)** | Median (95% CI) | 20.0 (8.6-nr) | | 13.5 (10.0-14.9) | | HR 0.66 (95% CI 0.31-1.39);  p-value 0.272 |
| **Progression (platinum-based chemo only; *n* = 81)** | Yes | 9 | 64.3 | 63 | 91.3 | 0.007 |
| **PFS (months) (platinum-based chemo only; *n* = 81)** | Median (95% CI) | 7.8 (7.0-10.1) | | 7.11 (5.9-9.0) | | HR 0.82 (95% CI 0.41-1.66);  p-value 0.585 |
| **Partial response (platinum-based chemo only; *n* = 81)** | Yes | 3 | 21.4 | 11 | 15.9 | OR 1.39 (95% CI 0.33-5.81);  p-value 0.653 |
| **Radiological reduction (platinum-based chemo only; *n* = 81)** | Yes | 9 | 64.3 | 25 | 36.3 | OR 3.02 (95% CI 0.91-10.04);  p-value 0.071 |
| **Died (platinum-based chemo only; *n* = 81)** | Yes | 8 | 53.3 | 58 | 78.4 | 0.043 |
| **OS from palliative therapy initiation (months) (platinum-based chemo only; *n* = 81)** | Median (95% CI) | 29.4 (7.9-26.7) | | 13.3 (11.0-16.4) | | HR 0.79 (95% CI 0.39-1.61);  p-value 0.527 |
